# Evaluation of Predisposing Factors and Coexisting Diseases in the Development of Chronic Thromboembolic Pulmonary Disease

**DOI:** 10.1111/crj.70147

**Published:** 2025-12-28

**Authors:** Ebru Sengul Parlak, Beyza Aybuke Aydin Uzun, Kubra Gungor, Eren Goktug Ceylan, Kubra Isik, Rabia Damla Kiziltas, Dina Serin, Umran Ozden Sertcelik, Serdal Bastug, Zeynep Hande Kocaer, Derya Sokmen, Izzet Selcuk Parlak, Ayşegül Karalezli

**Affiliations:** ^1^ Department of Chest Diseases Ankara Bilkent City Hospital Ankara Türkiye; ^2^ Department of Cardiology Ankara Bilkent City Hospital Ankara Türkiye; ^3^ Department of Radiology Ankara Bilkent City Hospital Ankara Türkiye

## Abstract

**Introduction:**

The objective of this study was to examine the development of chronic thromboembolic pulmonary disease (CTEPD) incidence, risk factors, and coexisting medical conditions following an episode of acute pulmonary embolism (PE).

**Materials:**

This retrospective, cross‐sectional study analyzed data from 722 patients diagnosed with PE. Group I (*n* = 663), consisting of individuals who did not develop CTEPD, and Group II (*n* = 59), comprising those who progressed to CTEPD. CTEPD were divided into two subgroups as chronic thromboembolic pulmonary hypertension (CTEPH, *n* = 23) and without pulmonary hypertension (PH) (*n* = 36). The groups were compared based on demographic features, comorbid conditions, risk factors, and initial systolic pulmonary artery pressure (sPAP) values.

**Results:**

CTEPD was observed in 59 patients (8.2%). Chronic obstructive pulmonary disease, coronary artery disease, and elevated baseline sPAP demonstrated a significant association with CTEPD (*p* = 0.003, *p* = 0.041, and *p* = 0.024, respectively). Immobilization was found to be significantly more prevalent in Group I (*p* = 0.032). In the multivariate logistic regression analysis, each 1 mmHg increase in baseline sPAP was associated with a 1.04‐fold elevation in the risk of CTEPD development (95% confidence interval [CI]: 1.02–1.05; *p* < 0.001). Additionally, a 1‐year decrease in age was linked to a 1.03‐fold increase in the probability of developing CTEPD (95% CI: 1.01–1.05; *p* = 0.003). No significant differences were found between patients with CTEPH and those with CTEPD without PH.

**Conclusion:**

These findings highlight the important role of comorbid conditions in the development of CTEPD. It is important to optimize the clinical management of patients with such comorbidities to reduce the risk of CTEPD development.

## Graphical abstract

In this cohort of 722 patients with acute pulmonary embolism, 8.2% developed chronic thromboembolic pulmonary disease (CTEPD). CTEPD was more frequently observed in patients with comorbidities such as COPD and coronary artery disease, and in those with higher baseline systolic pulmonary artery pressure, with younger age also showing an association. These findings suggest that comorbid conditions and baseline pulmonary pressure parameters may be relevant in the follow‐up and clinical management of patients after acute pulmonary embolism.

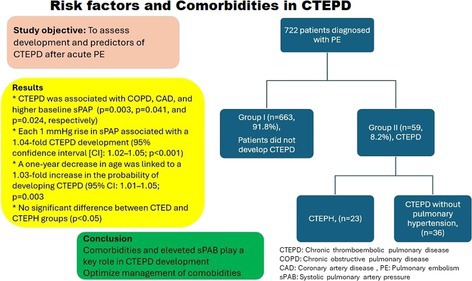

## 1 Introduction

Pulmonary hypertension (PH) is a pathophysiological condition encompassing various clinical disorders linked to cardiovascular and respiratory diseases [1]. Chronic thromboembolic pulmonary disease (CTEPD) without or with PH (CTEPH) is characterized by the presence of post‐thromboembolic fibrotic obstruction in the pulmonary artery after at least 3 months of effective anticoagulant use after acute pulmonary embolism (PE) and classified as Group 4 PH [1, 2]. The annual estimated incidence is approximately 5–6 cases/million people [2]. Almost 75% of cases have a history of acute PE [3].

CTEPH is a well‐known complication after acute PE [4]. Two main hypotheses have emerged to explain the development of CTEPH. The first suggests that local in situ thrombosis in small arteries leads to distal arteriopathy and subsequently to PH. According to the second hypothesis, emboli of systemic origin become trapped within the organized pulmonary vessels, and their dissolution process fails. Stabilized thrombotic lesions can lead to distal microvascular arteriopathy and elevated pulmonary pressures. A characteristic finding in CTEPH is organized thrombus. Additional vascular abnormalities include medial hypertrophy of the pulmonary arteriole muscular layer, concentric laminal internal fibroelastosis, and plexiform lesions [5]. CTEPH has been associated with many conditions and diseases. Thrombophilic disorders (elevated clotting factor VIII levels, antiphospholipid antibody syndrome), inflammatory bowel disease, cancer, ventriculo‐atrial shunts, splenectomy and chronic intravenous lines, and medical device‐related infections are well‐known risk factors [3].

Imaging techniques can identify characteristics such as chronic total occlusions, rings, slits, and webs. In certain cases, patients may exhibit symptoms despite having normal pulmonary hemodynamics at rest. After ruling out other potential causes of exercise limitation, these patients are diagnosed with chronic thromboembolic disease [3]. CTEPD without PH patients constitute a minority of those evaluated at CTEPH centers [1]. Approximately half of patients who experience an acute PE continue to show residual perfusion defects 1 year after the event; however, only a small proportion meet the hemodynamic diagnostic criteria for CTEPH [6]. To better characterize patients with persistent perfusion abnormalities but without PH at rest, the term CTEPD without PH has been introduced [2, 6].

Anatomically, CTEPH is associated with large‐vessel fibrotic obstruction and secondary small‐vessel microvasculopathy, whereas the clinical significance of CTEPD without PH remains uncertain. Recognition of CTEPD without PH has gained increasing importance, as it appears to be more prevalent yet frequently underdiagnosed [6]. Moreover, selected symptomatic patients with CTEPD without PH may be candidates for treatment options such as pulmonary endarterectomy or balloon pulmonary angioplasty, emphasizing the importance of proper identification and characterization of this condition [1].

The aim of this study was to evaluate the frequency, comorbidities, and factors associated with the development of CTEPD after acute PE.

## 2 Methods

This study was designed as a retrospective, cross‐sectional, single‐center study. After obtaining approval from the local ethics committee (Approval Number: E2‐22‐1514), a total of 722 patients aged 18 years and older who were diagnosed with PE between 2019 and 2022 were included in the study. Pregnant women and individuals with mental disabilities were excluded.

Patients were evaluated in two main groups: those who did not develop CTEPD (Group I, *n* = 663) and those who developed CTEPD (Group II, *n* = 59) after acute PE. In a subgroup analysis, CTEPD was further classified into CTEPH and CTEPD without PH.

CTEPD was diagnosed after at least 3 months of effective anticoagulant therapy, based on the presence of segmental or larger mismatched perfusion defects on ventilation–perfusion (V/Q) scintigraphy and/or organized thrombus, wall‐adherent clot, web, band, stenosis, or pouch‐like formations demonstrated on computed tomography pulmonary angiography (CTPA). All radiological evaluations were performed and reviewed by radiologists experienced in PE and chronic thromboembolic disease. V/Q scans and CTPA images were interpreted according to standard diagnostic criteria, and these imaging criteria were applied consistently across all patients to ensure diagnostic accuracy. Patients meeting these criteria were categorized according to hemodynamic findings: those with pre‐capillary PH confirmed by right heart catheterization were classified as CTEPH. The diagnosis of CTEPD without PH was established only after alternative causes of dyspnea were systematically excluded. All patients underwent comprehensive clinical and functional evaluation, including spirometry, echocardiography, and cardiology consultation to exclude left heart disease, relevant valvular abnormalities, or parenchymal lung diseases. As all patients were diagnosed before the release of the 2022 ESC/ERS Guidelines on PH, hemodynamic classifications were made according to the 2015 ESC/ERS recommendations (precapillary PH was defined as mean PAP ≥ 25 mmHg, pulmonary arterial wedge pressure ≤ 15 mmHg, and pulmonary vascular resistance [PVR] > 3 Wood units) for patients with CTEPH [7].

Patient data were retrospectively obtained from medical records. Demographic characteristics, comorbidities, risk factors, and baseline systolic pulmonary artery pressure (sPAP) were evaluated and compared between groups.

Factors associated with CTEPD development were evaluated using binary logistic regression. The analysis was conducted with 482 participants who had complete data. Patients with missing sPAP (mmHg) values (33%) were excluded. Baseline sPAP measurements were obtained from transthoracic echocardiography performed at the time of acute PE diagnosis. In a subset of patients, sPAP could not be assessed due to inadequate acoustic windows or the absence of a measurable tricuspid regurgitation jet; these individuals were therefore not included in the multivariable regression model. Multiple imputation was not feasible because echocardiographic assessments were retrospective, image acquisition was not standardized, and raw images were unavailable for review. Moreover, the mechanism of missingness was related to technical limitations, indicating that data were not missing at random and violating key assumptions required for valid imputation. For these methodological reasons, a complete‐case analysis was deemed the most appropriate approach.

### 2.1 Statistical Analysis

Statistical analyses were conducted using IBM SPSS Statistics software (Version 22.0; SPSS Inc., Chicago, IL, USA). For continuous variables with a normal distribution, comparisons between groups were made using the Student's *t*‐test, while the Mann–Whitney U test was applied to data that did not follow a normal distribution. Descriptive statistics for continuous variables were presented as means and standard deviations. Categorical variables were analyzed using the chi‐square (χ^2^) test. Independent predictors were identified using multivariable logistic regression analysis. The strength of association for each covariate was reported as an odds ratio (OR) with 95% confidence intervals (CIs). A *p*‐value of 0.05 or less was considered statistically significant.

## 3 Results

**FIGURE 1:**
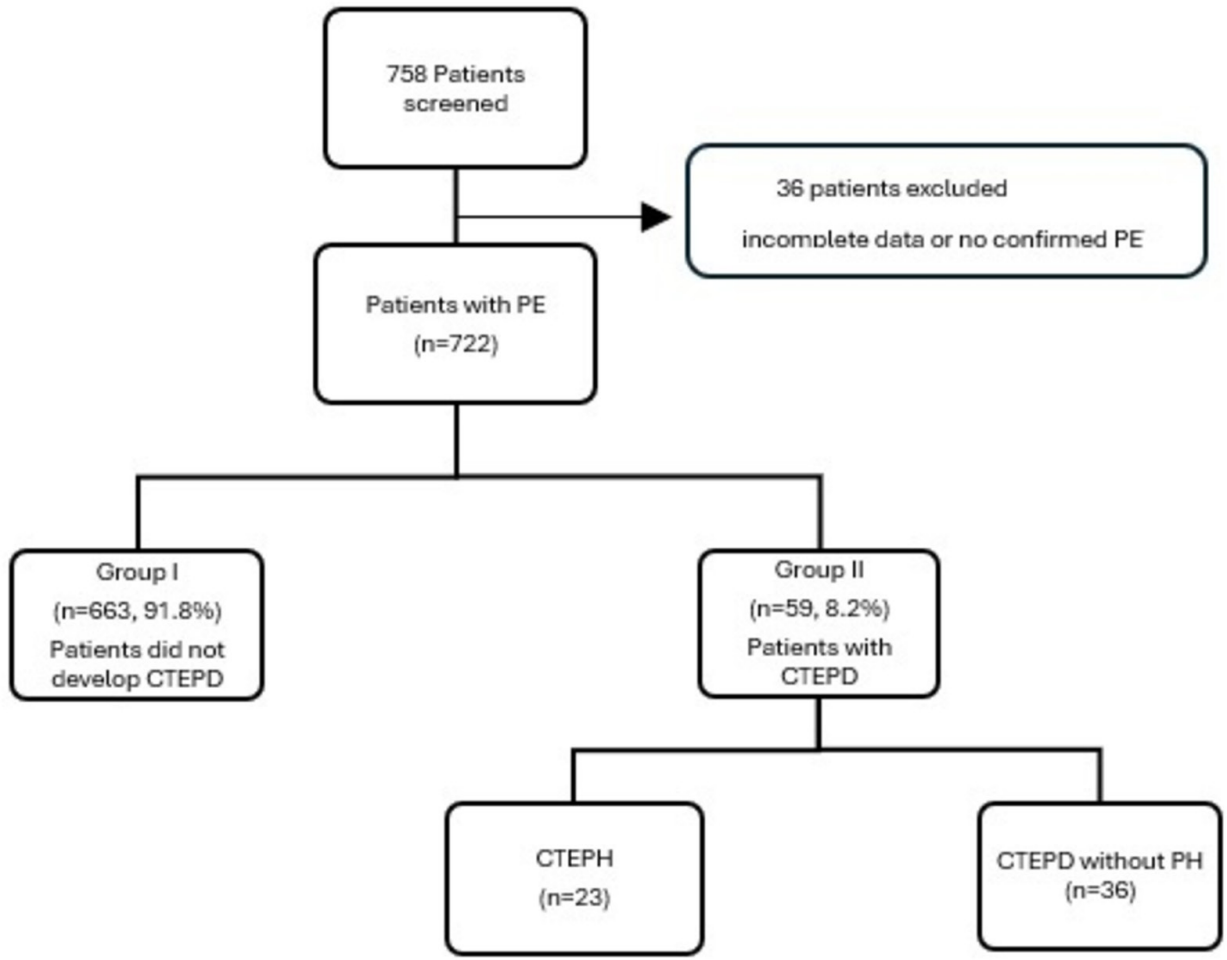
Flowchart of the study population. CTEPD: chronic thromboembolic pulmonary disease, CTEPH: chronic thromboembolic pulmonary hypertension, PE: pulmonary embolism, PH: pulmonary hypertension.

**FIGURE 2:**
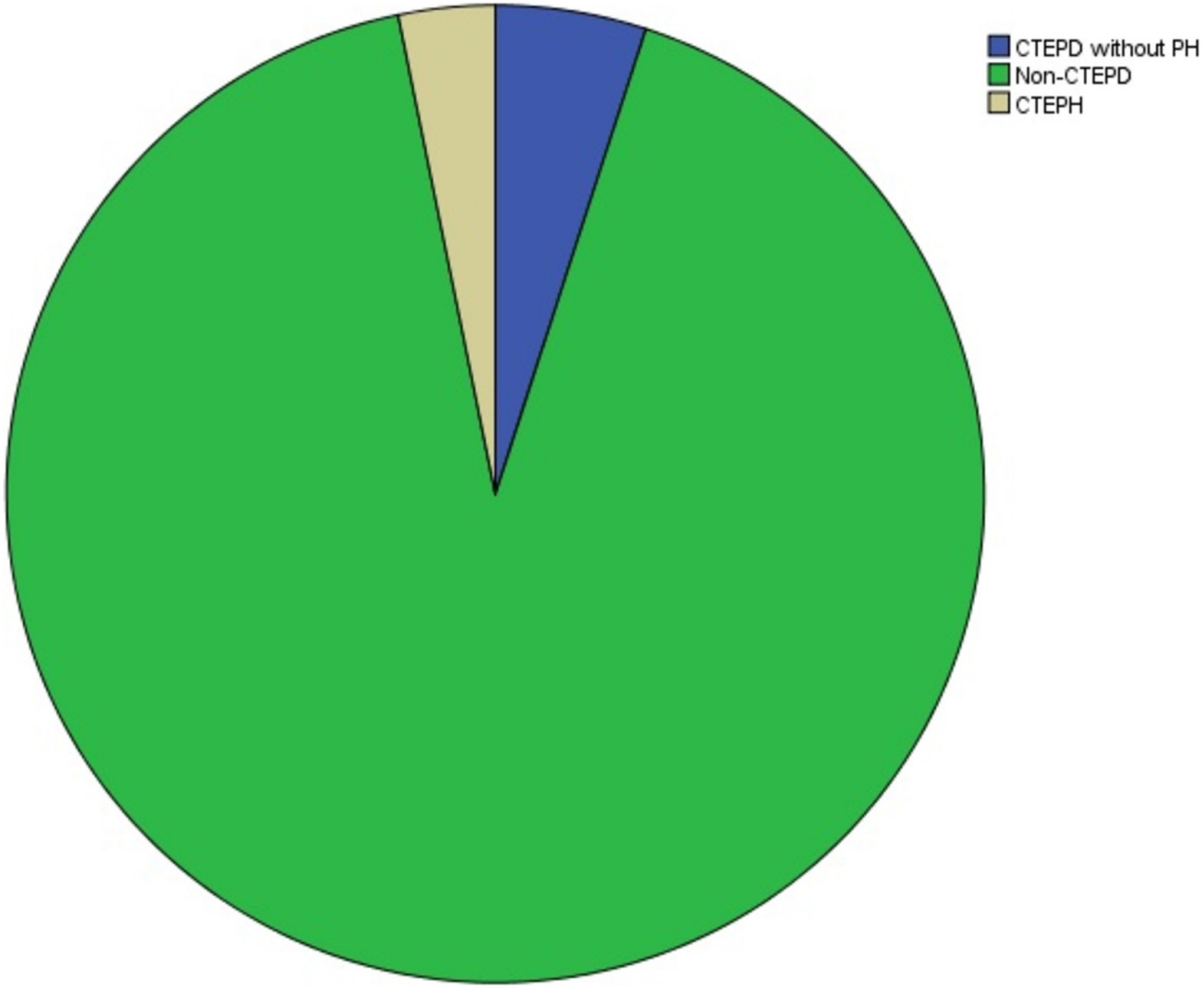
Distribution of patients according to chronic thromboembolic disease status. CTEPD: chronic thromboembolic pulmonary disease, CTEPH: chronic thromboembolic pulmonary hypertension, PH: pulmonary hypertension.

In this study, the medical records of 758 patients were retrospectively evaluated. A total of 722 patients with a confirmed diagnosis of PE based on CTPA and/or V/Q scintigraphy, and for whom complete clinical data were available, were included in the final analysis (Figure 1). The distribution of patients by chronic thromboembolic disease status is shown in Figure 2. The mean age of the patients was 62.15 ± 17.35 years. There were 359 females (49.7%) and 363 males (50.3%). Among the 722 patients, 59 (8.2%) were diagnosed with CTEPD after acute PE (Group II), while the remaining 663 (91.8%) comprised Group I. Comparison between the groups revealed no statistically significant differences in terms of age (*p* = 0.163) or gender (*p* = 0.174). When overall comorbidity status was evaluated for each group, comorbidities were present in 80.2% (*n* = 532) of patients in Group I and 81.4% (*n* = 48) in Group II. While no statistically significant difference was observed between the groups regarding overall comorbid conditions (*p* = 0.836), subgroup analysis demonstrated that chronic obstructive pulmonary disease (COPD) and coronary artery disease (CAD) were significantly more prevalent in Group II (*p* = 0.003 and *p* = 0.041, respectively). In Group II, antiphospholipid antibody syndrome was detected in one (1.7%) patient (*p* = 0.082). Table 1 presents demographic data, comorbid conditions, and associated risk factors of the study population. An evaluation of the sPAP (mmHg) values recorded at the time of hospital admission revealed significantly higher levels in Group II (*p* = 0.024). sPAP (mmHg) values were available in most patients (Table 1). sPAP could be calculated in 49 of 59 (83.1%) patients with CTEPD and in 433 of 663 (65.3%) patients without CTEPD. In the remaining patients, estimation was not feasible because of inadequate echogenicity or the absence of a measurable tricuspid regurgitation jet. Immobilization was observed at a higher rate in Group I (*p* = 0.032). Immobility was defined as bed rest for at least three consecutive days or marked reduction in ambulation, as well as long‐distance travel lasting more than 4 h within the 4 weeks preceding the acute PE event [3, 8]. Immobility was identified in 10 patients within the CTEPD group. Among them, only one patient (10%) had a history of surgery, which was gastrointestinal system. Except for one patient, all immobile patients in this group were older than 70 years; immobility was attributed to various non‐surgical causes. In the group without CTEPD development, immobility secondary to surgery was present in 54 of 197 patients (27.4%). The most frequent type of surgery was orthopedic (*n* = 27, 13.7%), followed by neurosurgical procedures involving the central nervous system (*n* = 12, 6.1%).

**TABLE 1:** Baseline demographic data, comorbidities, and risk profiles of the patients.

Variables	Non‐CTEPD^a^ (*n* = 663)	CTEPD^a^ (*n* = 59)	*p*
Age (years)	62.40 ± 17.39	58.35 ± 16.38	0.163
Gender, *n* (%)			0.174
Female	335 (50.5)	24 (40.7)	
Male	328 (49.6)	35 (59.3)	
Smoking status, *n* (%)			0.209
Smoker	187 (28.2)	15 (25.4)	
Non‐smoker	339 (51.1)	26 (44.1)	
Ex‐smoker	137 (20.7)	18 (30.5)	
Comorbidities and risk factors, *n* (%)			
Diabetes mellitus	158 (23.8)	11 (18.6)	0.367
Hypertension	323 (48.7)	30 (50)	0.754
Coronary artery disease	196 (29.6)	25 (42.4)	0.041
Heart failure	89 (13.4)	12 (20.3)	0.142
Atrial fibrillation	79 (11.9)	7 (11.9)	0.991
COPD^b^	111 (16.7)	19 (32.2)	0.003
Bronchial asthma	60 (9)	6 (10.2)	0.775
Bronchiectasis	18 (2.7)	2 (3.4)	0.762
Malignancy	100 (15.1)	6 (10.2)	0.307
Collagen tissue disease	28 (4.2)	3 (5.1)	0.734
Hypothyroidism	58 (8.7)	7 (11.9)	0.423
Stroke	59 (8.9)	5 (8.5)	0.912
Chronic renal failure	46 (6.9)	5 (8.5)	0.598
Immobilization	197 (29.7)	10 (16.9)	0.032
sPAP^c^ (mmHg)	35.6 ± 13.22	44.73 ± 23.56	0.024
Thrombolytic therapy	45 (6.8)	5 (8.5)	0.592

^a^ CTEPD: chronic thromboembolic pulmonary disease.

^b^ COPD: chronic obstructive pulmonary disease.

^c^ sPAP: systolic pulmonary artery pressure (sPAP available in 49/59 [83.1%] CTEPD and 433/663 [65.3%] non‐CTEPD patients; not measurable in others due to absent TR jet or poor echogenicity.).

All acute PE cases were treated with medical therapy. Endarterectomy was performed in four (6.7%) of the patients with CTEPH.

Binary logistic regression identified the factors associated with the development of CTEPD. A total of 482 patients, corresponding to 67% of the overall cohort (722 patients), had sufficient echocardiographic data and were included in the multivariable analysis. The remaining 33% lacked measurable sPAP values and were therefore not analyzed, as these parameters could not be reliably obtained. Univariate analysis revealed that COPD, CAD, and sPAP (mmHg) were statistically significantly associated with CTEPD development. In multivariate analysis, age and sPAP (mmHg) remained statistically significant factors associated with CTEPD when female gender, COPD, and CAD were controlled. Younger age was independently associated with increased odds of CTEPD (95% CI: 1.01–1.05; *p* = 0.003). Similarly, each 1 mmHg increase in sPAP increased the risk of CTEPD by 1.04 times (95% CI: 1.02–1.05; *p* < 0.001). The results of the binary logistic regression analysis of factors associated with the risk of CTEPD are shown in Table 2.

**TABLE 2:** Binary logistic regression outcomes for factors linked to chronicity in the overall study population.

	Univariate analysis		Multivariate analysis	
	OR (95% CI)	*p*	OR (95% CI)	*p*
Age (years)	0.99 (0.98–1.01)	0.197	0.97 (0.95–0.99)	**0.003**
Female gender	0.67 (0.39–1.15)	0.149	0.73 (0.38–1.39)	0.338
COPD^a^	2.36 (1.32–4.23)	**0.004**	1.84 (0.89–3.78)	0.099
CAD^b^	1.75 (1.02–3.01)	**0.043**	1.81 (0.93–3.54)	0.078
sPAP^c^ (mmHg)	1.03 (1.02–1.05)	**< 0.001**	1.04 (1.02–1.05)	**< 0.001**

*Note:* Hosmer–Lemeshow test *p*‐value = 0.480. Regression analysis included 482/722 (66.8%) patients with complete covariate data. Bold numbers indicate statistically significant results (*p* < 0.05).

Abbreviations: CI: confidence interval, OR: odds ratio.

^a^ COPD: chronic obstructive pulmonary disease.

^b^ CAD: coronary artery disease.

^c^ sPAP: systolic pulmonary artery pressure.

Patients diagnosed with CTEPD were further classified into two subgroups: CTEPH (*n* = 23, 3.2%) and CTEPD without PH (*n* = 36, 5%). No statistically significant differences were identified between these subgroups in terms of age, gender, risk factors, or comorbidities (*p* < 0.005) (Table 3).

**TABLE 3:** Comparison of demographics, risk factors, and comorbidities between CTEPH and CTEPD without PH subgroups.

Variables	CTEPD^a^ without PH^b^ (*n* = 36)	CTEPH^c^ (*n* = 23)	*p*
Age (years)	59.41 ± 17.46	59.26 ± 15.48	0.972
Gender, *n* (%)			0.847
Female	15 (41.7)	9 (39.1)	
Male	21 (58.3)	14 (60.9)	
Comorbidities and risk factors, *n* (%)			
Diabetes mellitus	9 (25)	2 (8.7)	0.174
Hypertension	19 (52.8)	11 (47.8)	0.711
Coronary artery disease	14 (38.9)	11 (47.8)	0.498
Heart failure	5 (13.9)	7 (30.4)	0.185
Atrial fibrillation	2 (5.6)	5 (21.7)	0.098
COPD^d^	9 (25)	10 (43.5)	0.138
Bronchial asthma	4 (11.1)	2 (8.7)	1.000
Bronchiectasis	1 (2.8)	1 (4.3)	1.000
Malignancy	5 (13.9)	1 (4.3)	0.389
Collagen tissue disease	3 (8.3)	0 (0)	0.274
Hypothyroidism	4 (11.8)	3 (13)	1.000
Stroke	1 (2.8)	4 (17.4)	0.070
Chronic renal failure	3 (8.3)	2 (8.7)	1.000
Surgery within the last 4 weeks	4 (11.8)	1 (4.2)	0.639
Immobilization	6 (16.7)	4 (16.7)	1.000

^a^ CTEPD: chronic thromboembolic pulmonary disease.

^b^ PH: pulmonary hypertension.

^c^ CTEPH: chronic thromboembolic pulmonary hypertension.

^d^ COPD: chronic obstructive pulmonary disease.

## 4 Discussion

In this study, the rate of CTEPD was 8.2%. Of this, 3.2% comprised CTEPH cases, and 5% comprised CTEPD without PH. CTEPD development was more common among patients with CAD and COPD and less common in the immobilized group. Although COPD and CAD were more common in CTEPD, they did not remain independent predictors after multivariable adjustment. The sPAP value at hospital admission was higher in Group II. Additionally, a negative correlation was found between age and CTEPD. Age and sPAP were found to be statistically significant factors associated with CTEPD development.

Chronic thromboembolic PH accounts for approximately 20% of patients referred to major PH centers, as reported [6, 9]. A multicenter study showed that the cumulative incidence of CTEPH within 2 years after acute PE was found to be 0.79% [10]. A multicenter study conducted in Germany calculated the incidence of CTEPH as 5.7 per million adults in 2016 [11]. In a systematic review and meta‐analysis, the incidence of CTEPH was reported as 0.6% of all patients with acute PE and 3.2% in survivors [12]. The cumulative incidence of CTEPH within the first 2 years after acute PE has been reported to range between 0.1% and 11.8% [6]. In this study, the rate of CTEPH was 3.2% after acute PE, which is consistent with the findings in the literature. Generally, focus is placed on the development rates of CTEPH rather than CTEPD. In this study, the development of CTEPD, including not only CTEPH but also CTEPD without PH, was reported.

It has been shown that 30%–50% of patients continue to exhibit persistent perfusion defects 1 year after the diagnosis of acute PE [13, 14, 15]. There is no consensus in the literature regarding the clinical significance of persistent perfusion defects after acute PE [6]. While some studies have demonstrated an association between residual thrombus and impaired functional capacity or exercise tolerance [16, 17], a more recent study found no significant relationship between exercise capacity and the presence of perfusion defects [18].

Currently, CTEPH is generally diagnosed at an advanced stage of the disease, when more than 50% of the pulmonary vascular bed is obstructed, small‐vessel arteriopathy is established, and signs of right heart failure are evident [13]. Anatomically, in CTEPH patients, the increase in PVR results from both the obstruction of pulmonary arteries by unresolved, organized fibrotic clots and the development of a secondary microvasculopathy in both obstructed and non‐obstructed regions. This secondary microvasculopathy predominates in non‐obstructed lung areas and arises from flow redistribution, increased pressure, and shear stress [2, 6]. Considering the concept of the “pyramid of complications,” CTEPD without PH is believed to occur more frequently than CTEPH [6, 13]. In our cohort, CTEPD without PH was observed more frequently than CTEPH (5% vs. 3.2%), consistent with recent literature indicating that CTEPD without PH is a more prevalent yet often under‐recognized phenotype within the CTEPD spectrum. Furthermore, systematic post‐PE follow‐up of patients may have increased the identification of CTEPD without PH.

In this study subgroup analysis, no significant differences were found between CTEPD without PH and CTEPH in terms of age, sex, or major comorbidities. These findings indicate that both entities share a broadly similar clinical background, yet likely diverge in their pathophysiological mechanisms. While CTEPD without PH and CTEPH may coexist within the spectrum of chronic thromboembolic disease, current evidence suggests that CTEPD without PH does not necessarily represent an early or precursor stage of CTEPH [2], a distinct phenotype [19] characterized by persistent mechanical obstruction but probably without pulmonary vascular remodeling severe enough to cause PH. The lack of clear demographic or comorbidity‐based differences between these groups highlights that hemodynamic and imaging assessments remain more reliable than baseline clinical features for distinguishing disease phenotypes. According to current recommendations, further evaluation for CTEPH is advised in patients who present with persistent dyspnea, functional limitation, or established CTEPH risk factors following an episode of acute PE [3]. Implementing periodic and structured post‐PE follow‐up visits that include systematic symptom assessment and functional evaluation may facilitate the early identification of the CTEPD without PH phenotype, as well as associated exercise intolerance and perfusion abnormalities. Recognizing CTEPD without PH as a distinct clinical entity highlights the need for individualized long‐term monitoring strategies to ensure timely diagnosis and appropriate management.

Although some studies have suggested a link between CTEPH and advanced age [20, 21], the present study found a negative correlation between age and CTEPD. However, this study specifically analyzed the relationship between CTEPD and age, rather than focusing solely on CTEPH. The younger age observed in this study can therefore be attributed to the combined evaluation of CTEPH and CTEPD without PH. Moreover, a larger proportion of the cohort was classified as having CTEPD without PH, and the inclusion of these patients may have shifted the overall age distribution toward younger individuals. Systematic follow‐up after acute PE, along with referring younger and more symptomatic individuals to tertiary centers for further evaluation, may have introduced referral bias. In older individuals, symptoms are often insidious or progressive, which may result in diagnosis at a more advanced and hemodynamically significant stage of the disease. The retrospective design of the study may have contributed to misclassification by leading to a higher detection rate of milder or subclinical cases, particularly among younger patients. Age association should be interpreted with caution due to the retrospective design and potential referral bias.

Although immobilization is a well‐known risk factor for acute PE, it is not considered a predisposing factor for CTEPD. Immobilization is regarded as a weak and usually transient risk factor for acute PE [3]. This may explain its higher occurrence in the group without CTEPD development in the present study. The lower frequency of immobilization among patients with CTEPD may be attributed to the fact that most immobile patients were elderly, possibly leading to fewer hospital visits or a diminished perception of dyspnea due to limited exertion. In addition, the higher presence of inflammatory comorbidities such as COPD and CAD in the CTEPD group supports the notion that underlying inflammatory mechanisms, rather than physical inactivity, may play a more significant role in the pathogenesis of CTEPD.

PE of unknown cause, diagnostic delays of over 2 weeks, and right ventricular (RV) dysfunction at the time of PE are independent predictors of CTEPH [22]. Baseline sPAP > 60 mmHg has been associated with CTEPH [13]. In the current study, sPAP was found to be higher at baseline in Group II, with each 1 mmHg increase in sPAP raising the risk of CTEPD by 1.04 times.

Venous thromboembolism (VTE) and atherothrombosis are generally considered mechanistically different entities. Venous thrombus consists mainly of erythrocytes and fibrin in low‐flow vessels, called red thrombus. Arterial thrombus, or white thrombus, is composed mainly of platelets and contains little fibrin or red cells. Emerging evidence suggests that atherosclerosis and VTE may share a similar pathogenesis [23]. Common mechanisms initiating thrombus formation in both veins and arteries, such as endothelial activation, platelet aggregation, and leukocyte involvement, have been identified [24]. Recent research has increasingly supported the idea that atherosclerosis and VTE may share a common etiology driven by inflammatory processes [23, 24, 25]. Atherosclerosis is considered a chronic inflammatory disease, while the pathophysiology of VTE is associated with endothelial damage, venous stasis, and hypercoagulability [26].

Some previous prospective studies found no association between subclinical atherosclerosis assessed by carotid ultrasonography and the risk of VTE [27, 28]. In contrast, a cohort study including consecutive autopsies found an increased VTE in patients with atherothrombosis [29]. Furthermore, prospective cohort studies have shown an association between myocardial infarction and VTE [30, 31].

Prospective studies showed that patients with unprovoked PE had more cardiovascular events and atherothrombotic complications compared to those with secondary PE [32, 33]. Subjects with venous thromboembolic events had a higher risk of fatal cardiac infarction and stroke than the controls [34, 35]. A meta‐analysis showed that patients with VTE had a higher risk of arterial cardiovascular events [36]. In a study comparing acute PE survivors without CTEPH to those with CTEPH, it was shown that the incidence of CAD was higher in the CTEPH group [25]. In this study, CAD was observed at a higher rate among the patients with CTEPD; nevertheless, it did not retain statistical significance after adjustment for other covariates in the multivariable analysis. This may indicate overlapping risk profiles or shared pathophysiological mechanisms between arterial and venous thrombosis.

Restrictive pulmonary defects are observed in nearly 20% of patients diagnosed with CTEPH. This defect is characterized by a reduction in lung volume to less than 80% of the predicted value, likely due to parenchymal scarring following lung infarction. Additionally, patients with CTEPH may experience mild decreases in forced expiratory volume (FEV)_1_. Although hypoxemia in CTEPH cases is primarily due to V/Q mismatch associated with pulmonary vascular disease, other lung function disorders should not be ignored [37]. Notably, patients with CTEPH frequently exhibit obstructive ventilatory impairment, even if they have no history of smoking [38]. COPD is characterized by a reduction in the effective alveolar surface area and changes in the alveolar‐capillary membrane due to emphysematous changes in the lung tissue [39]. A significant factor contributing to flow limitation in CTEPH is inflammatory thrombosis. Inflammatory mediators have been reported to be produced from blood clots and remodeled pulmonary arteries [38]. Chronic embolism leads to increased PVR and impaired gas exchange across the alveolar‐capillary membrane. Oxygen tension decreases due to V/Q mismatch, abnormal airways and blood bypass, resulting in hypoxemia [39]. COPD is thought to be associated with VTE due to decreased mobility, systemic inflammation, tobacco use, and impaired venous return in patients. However, VTE is often underdiagnosed in COPD cases because its symptoms mimic those of COPD exacerbations. Autopsy studies have reported PE in 28%–51% of COPD cases [40]. In the current study, the rate of COPD was found to be significantly higher in the CTEPD group than in patients who did not develop CTEPD, which may be related to V/Q mismatch, chronic hypoxia, inflammation, and pulmonary vascular remodeling due to capillary loss in COPD. However, this association did not remain statistically significant in the multivariable regression model, suggesting that COPD may act as a contributing comorbidity rather than an independent predictor of CTEPD. It is plausible that the observed relationship reflects shared pathophysiological mechanisms—such as endothelial dysfunction and chronic inflammation—rather than a direct causal link.

In summary, this study offers important insights into the frequency and associated clinical characteristics of CTEPD following an episode of acute PE. The study identified a notable association between younger age and CTEPH, contrary to earlier reports suggesting a predominance in older populations. In our cohort, the inclusion of patients with both CTEPH and CTEPD without PH, along with close post‐PE follow‐up and referral patterns to tertiary centers, may help explain this observed pattern. The higher prevalence of immobilization in the non‐CTEPD group compared with CTEPD patients may also indicate the potential influence of underlying inflammatory processes, although this relationship should be interpreted within an associative framework. The observed relationship between elevated baseline sPAP and CTEPH is consistent with existing literature. Although CAD did not remain an independent variable in the multivariable model, its higher frequency among patients with CTEPD may suggest overlapping mechanisms described in studies of arterial and venous thrombosis. Similarly, COPD was more common in patients with CTEPD, but the lack of statistical significance after adjustment limits interpretability; nevertheless, its coexistence underscores the need for clinical awareness, as overlapping symptoms may contribute to delayed recognition. Taken together, these findings highlight patient characteristics that may warrant closer monitoring after acute PE, particularly in individuals with comorbidities such as COPD, CAD, and elevated baseline sPAP. Efforts should focus on refining diagnostic criteria and developing targeted prevention and treatment approaches to mitigate the burden of CTEPD in high‐risk populations.

This study has several limitations, including its retrospective design, lack of extended prospective follow‐up, and single‐center design. Because the patients were not followed prospectively, the median duration between acute PE diagnosis and CTEPD development could not be assessed. Patients fulfilling the diagnostic criteria were identified retrospectively from medical records. Because the data could not be reclassified according to the 2022 criteria, the findings may not fully reflect the current definitions.

Another limitation of this study is that patients with missing sPAP data were excluded from the multivariable regression model. A complete‐case analysis was used because missing sPAP values resulted from technical echocardiographic limitations, and the pattern of missingness was not random, making multiple imputation inappropriate for this dataset. This may have reduced the sample size for the regression analyses and slightly affected the precision of the estimates, which should be considered when interpreting the findings. In addition, a comprehensive evaluation of broader risk determinants—such as genetic factors, lifestyle habits, and environmental exposures—was not included.

The main findings and clinical implications are illustrated in the Graphical Abstract.

## Author Contributions

**Ebru Sengul Parlak:** formal analysis, investigation, methodology, writing – original draft, writing – review and editing. **Beyza Aybuke Aydin Uzun:** data curation, writing – original draft. **Kubra Gungor:** data curation, investigation, writing – original draft. **Eren Goktug Ceylan:** data curation, writing – original draft. **Kubra Isik:** data curation, writing – original draft. **Rabia Damla Kiziltas:** data curation, writing – original draft. **Dina Serin:** data curation, writing – original draft. **Umran Ozden Sertcelik:** formal analysis, writing – original draft. **Serdal Bastug:** methodology, writing – original draft. **Zeynep Hande Kocaer:** data curation, writing – original draft. **Derya Sokmen:** data curation, writing – original draft. **Izzet Selcuk Parlak:** methodology, writing – original draft. **Ayşegül Karalezli:** supervision, writing – original draft.

## Funding

The authors have nothing to report.

## Ethics Statement

The study was approved by the Ethics Committee of Ankara Bilkent City Hospital (Approval Number: E2‐22‐1514).

## Conflicts of Interest

The authors declare no conflicts of interest.

## Data Availability

The data that support the findings of this study are available upon request from the corresponding author. The data are not publicly available due to privacy or ethical restrictions.
